# The Effect of Diltiazem on Microcirculation of Transplanted Tissue Flaps: Experimental Study on Rabbits

**DOI:** 10.29252/wjps.10.2.61

**Published:** 2021-05

**Authors:** Mahammad M Davudov, Jamal Musayev, Adalat Hasanov, Hamid Reza Fathi

**Affiliations:** 1 Department of Oral and Maxillofacial Surgery, Azerbaijan Medical University, Baku, Azerbaijan.; 2 Department of Pathology, Azerbaijan Medical University, Baku, Azerbaijan.; 3 Department of Plastic, Reconstructive and Aesthetic Surgery, Tehran University of Medical Sciences, Tehran, Iran.

**Keywords:** Diltiazem, Experiment, Histology, Microcirculation, Pedicle flap

## Abstract

**BACKGROUND:**

The aim of the presented study was to investigate the inhibitory effect of diltiazem on the microcirculation of the tissue flaps created in the delay phenomenon applied rabbits.

**METHODS:**

The experiment was performed in Central Research Laboratory of Azerbaijan Medical University, Baku, Azerbaijan in 2018. Ischemia model on the flaps were formed in 30 rabbits for experiment. The subjects were divided into three groups: I (control) group - consists of 10 rabbits who underwent ischemia model, but no medication had been used during the duration of the experiment; II (comparative) group - consists of 10 rabbits who underwent ischemia model, and during 14 d, with a daily dose of 60 mсg nitroglycerin had been applied; III (main) group - consists of 10 rabbits who underwent ischemia model, and during 14 d, with a daily dose of 45 mg diltiazem had been applied.

**RESULTS:**

There was statistically significant difference between the control and the main groups when comparing the mean values of endothelial hyperplasia (*P*=0.001). However, we found a statistically significant difference when compared the mean values of the arterial vessel wall thickening in the main and the control groups (*P*=0.022); and the mean values of thrombosis in the main and the comparative groups (*P*=0.001).

**CONCLUSION:**

With prescription of diltiazem, endothelial hyperplasia on microcirculatory system, the thickening of arterial vessel walls, the thrombosis in vessel’s passage was rarely seen by statistical difference. The main achievement of our study was to discover the correlation between diltiazem and endothelial hyperplasia.

## INTRODUCTION

Reconstructive interventions for congenital abnormalities or following extensive surgical treatment of malignant tumors of the head and neck region are often performed with free tissue flaps. To avoid postoperative complications, the flaps are controlled pre-and postoperatively, and various medicines are used for optimal regenerative processes in the transplanted tissues.

The most common cause of early complications in the pedunculated and free flaps is the formation of thrombosis in the peduncle vessels and the anastomosis^1^. The main cause of complications is the narrowing of vessels lumen by intimal hyperplasia^2^.

Many medicines have been used to prevent intimal hyperplasia. Medications such as calcium channel blockers, beta-blockers, and angiotensin converter enzyme inhibitors have yielded significant results in delaying intimal hyperplasia. The cause of the limitations in clinical application of such drugs is that their mechanism of action is not sufficiently known^3^. Diltiazem is a calcium channel blocker belonging to the benzothiazepine group and is used in cardiovascular surgery to prevent contraction of smooth muscle cells of vessel wall by preventing the entry of calcium ions into the muscle cells. 

The presented study aimed to investigate the inhibitory effect of diltiazem on the microcirculation of the pedunculated tissue flaps created in the delay phenomenon applied rabbits.

## MATERIALS AND METHODS

**Fig.1:**
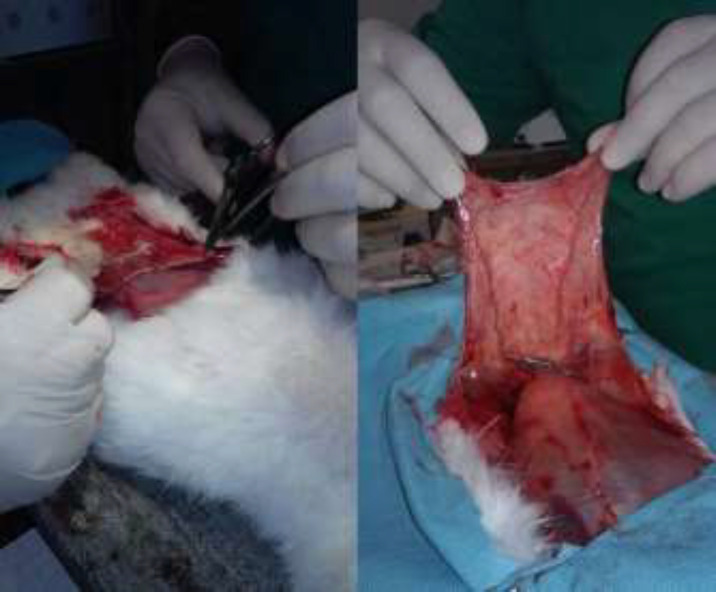
Preparation of experimental tissue flap in rabbit

Experiment was performed in Central Research Laboratory of Azerbaijan Medical University, Baku, Azerbaijan in 2018. Thirty 1-2 yr old rabbits with a weight of 2,1-3,0 kg were used for the experiment. The ischemia model on the flaps were formed in all subjects. For this purpose, 1.0 gr of calypsol solution was applied to the subjects via intramuscular injection. The hairin the abdominal skin of the subjects has been cleared and the crescent-shaped section which encompasses skin and subcutaneous tissue was applied (Figure 1). The right and left branches of the arteria epigastrica inferior were dissected. The left vascular pedicle was ligated and the blood flow was stopped. An ischemia model has been created for 1 hour on the right vascular pedicle. For this purpose, the intermittent clamping method was used and the vascular pedicle was tightened 3 times with 10 min intervals and 10 min each. Following the successful application of the ischemia model, the skin margins were adapted to each other and they were mended with silk thread No 4. Surgical wounds were cleaned with antiseptic solutions. 0.3 gr ceftriaxone was applied via intramuscular injection to prevent infection complications.

The Ethics Committee of Azerbaijan Medical University approved the study in accordance with the tenets of the Helsinki Declaration and national ethical guideline for medical research. Ethics Approval Code: AZ. AMU.06072018.05.

The ischemia model-created skin was removed with surrounding healthy tissues by excision after 2 wk (14 d) upon completion of the experiment. For this purpose, 1.0 gr of calypsol solution was applied to the subjects via intramuscular injection. Euthanasia was performed in all subjects via injection of 20.0 mg air to auricular vein.

The experimental animals were divided into three groups, each containing 10 rabbits after the creation of the ischemia model in the skin flap:

First group (control group) - consists of 10 rabbits who underwent ischemia model, but no medication were used in the duration of the experiment.

Second group (comparative group) - consists of 10 rabbits who underwent ischemia model, and during 14 d, a daily dose of 60 mсg Nitroglycerin had been applied.

Third group (main group) -consists of 10 rabbits who underwent ischemia model, and during 14 d, a daily dose of 45 mg diltiazem was applied.

Histologically, some morphological parameters of the microcirculation such as endothelial hyperplasia, thickening of the arterial vessel wall, thrombosis, congestion and edema were evaluated with a semiquantitative method of 4 degrees [0-3] for their intensity. 

Statistical calculations were made on the results obtained after this evaluation. In the statistical evaluation, the average size of various parameters, their mean standard deviation, P-value between the different groups were calculated using SPSS (ver.15.0, Chicago, IL, USA). The variance analysis (ANOVA test) was used to measure the *P*-value and the significance threshold was accepted as *P*≤0.05.

## Results

**Fig. 2:**
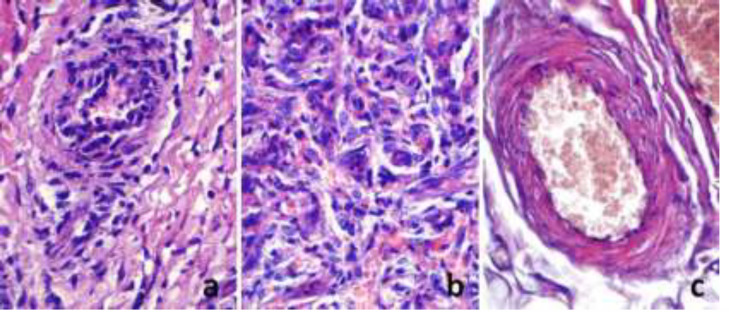
a: Papillary endothelial hyperplasia in the vessel and collagen proliferation in surrounding tissue in the sample from control group; b: Total flat hyperplasia in the vascular endothelium and collagen proliferation in the surrounding tissue in the sample from comparative group; c: Focal flat endothelial hyperplasia in the sample from main group (stain: hematoxylin-eosin, magnification: x100).

Endothelial hyperplasia, thickening in the arterial vessel wall, and thrombosis were significantly lower in the main group. Endothelial thickening was seen mostly in control group, less in comparison group, and the least in the main group (Table 1). General hyperplasia, swelling, papillary proliferation of endothelial layer were evident in some samples of the control group (Figure 2a).

Although the findings are usually seen in the veins of the suture side of the tissue flap, various reactive lesions were observed in the veins of the central part of the flap. In this group, endothelial lesions mainly were seen in arterial vessels. Endothelial lesions were more prominent in the areas of capillary proliferation (granulation tissue) in surgical section side of the flap. Lumen obstruction was seen in some vessels due to endothelial hyperplasia. Endothelial hyperplasia was seen just in some samples of the comparative group (Figure 2b); it was total and flat type hyperplasia, and papillary proliferation has not been observed in any case. Vascular obliteration due to endothelial hyperplasia did not appear. Focal or total flat endothelial hyperplasia was identified in only a few of the main group samples (Figure 2c). There was a statistically significant difference between the control and the main groups when comparing the mean values of endothelial hyperplasia (*P*=0.001). However, there was no statistically significant difference between the comparative and the main groups when comparing the same parameter (*P*=0.096). 

**Table 1:** The average values ​​of the microcirculatory parameters in different groups

**Microcirculatory parameters**	**Control group**	**Comparative group**	**Main group**
Endothelial hyperplasia	1.8±0.788	0.9±0.737	0.4±0.516
Thickening of the arterial vessel wall	1.2±1.032	0.4±0.516	0.3±0.483
Thrombosis	1.6±0.699	0.6±0.699	0.4±0.516
Congestion	0.2±0.699	1.0±0.942	2.2±0.788
Edema	0.5±0.707	0.9±0.737	1.8±0.918

**Fig. 3:**
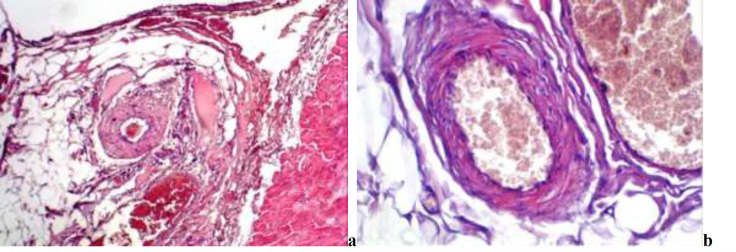
**a-**Thickening of the arterial vessel wall in the control group (stain: hematoxylin-eosin, magnification: x100). b-Vascular wall in the main group; normal vascular wall and normal endothelial layer (stain: hematoxylin-eosin, magnification: x100).

In the arterial vessels, the middle layer of the wall (media) was thickened mainly in the control group besides endothelial lesions. Although not as intensive as endothelial hyperplasia is, some thickening in the arterial vessel wall has been noticed in certain samples. The thickening of the vessel wall was caused mainly by sclerotic changes (Figure 3).

Several samples demonstrated elastosis and hyalinosis in the vessel wall. Lesions of the vessel wall also occurred mainly in the surgically sectioned part and partly in the central part of the skin. Samples of comparative and control groups illustrated minimal changes in the vessel wall. Thickening of medial layer was revealed only in few samples and thickening caused by sclerotic changes in all samples. We found a statistically significant difference (*P*=0.022) when comparing the mean values of the arterial vessel wall thickening in the main and the control groups.

In the control group, non-occlusive micro thrombosis in some samples, and total occlusive thrombotic mass in one sample were detected. Thrombotic masses were mainly hyaline type and they were closely related to endothelial cells. In the comparative group, thrombosis has been recorded in just a few samples. Relevant changes were not recorded in the main group. The statistically significant difference was detected when comparing the mean values of thrombosis in the main and the comparative groups (*P*=0.001).

## Discussion

The use of various medicaments for regeneration of transplanted tissue in rehabilitation surgery is essential^4^. In free tissue transplantation, treatment with medicaments is one of the most common options. Aspirin is used as an inhibitor of endothelial cyclooxygenase to alleviate platelet aggregation within 2-3 wk postoperatively. Dextran is infused to normalize the rheological properties of the blood, to reduce its self-esteem. Heparin is a part of postoperative protocols and is administered at least 3 d after surgery, but the use of heparin increases the likelihood of hematomas. Although the latter is associated with anastomotic problems, the timely detection of the problem and the appropriate measures can prevent more severe complications such as transplant necrosis^5^^,^^6^. The standard and commonly accepted method for selecting the transplant is a clinical assessment, so the color, osmotic pressure, capillary refill, the Doppler ultrasound of tissue peduncle for the first 3 d and daily prick test can be helpful for selection. The healthy flaps are pink, have high temperature and small amounts of edema and its capillary resuscitation should be within 1-3 seconds. About 1-3 drops of red blood should flow from the area of prick test^7^^,^^8^.

Thrombosis of vein anastomosis is the cause of over 90% of the flap surgery failures and haematoma formation is the main finding of such complications. In such cases, the patient is immediately taken to the operating room, the area exposed to operative intervention is re-opened, the thrombectomy is performed, and the anastomosis supposed to be re-evaluated. This problem can be solved in 50% of the available techniques^9^. Timely detection and elimination of possible complications in transplant operations remains a matter of urgency, and new investigations and medications are deemed necessary in this direction.

The relationship between diltiazem and thrombosis has been studied in many studies and various results have been obtained. In some research studies, diltiazem was not able to prevent thrombosis, but others were found to be contradictory. Diltiazem is unable to prevent thrombosis when determining the dose that eliminates hemodynamic disorders on different animals, especially in the experiments with the creation of a coronary thrombosis model^10^^-^^12^. In the experimental coronary microemboli model formed on mice, coronary thrombosis symptoms were partially eliminated by intravenous diltiazem detection^13^. In our study, diltiazem in therapeutic dose was able to prevent thrombosis of tissue flaps in rabbits.

The fact that diltiazem prevents endothelial damage due to hypoxia in the vessel wall, identified in our study, it is also determined in previous studies. An experimental study on mice has shown that diltiazem significantly reduced the endothelial apoptosis associated with hypoxia, levels of angiotensin I and endothelin II^9^.

Intimal hyperplasia is a universal response mechanism for arterial hemodynamic stresses and is usually part of the expected healing process after any injury. The intimal thickening occurs mainly due to the proliferation or hyperplasia of endothelial cells. Endothelin-1 (ET-1), synthesized in endothelial cells, is the most powerful vasoconstrictor factor. This peptide creates vasoconstriction through endothelin A and B receptors (ET-A and ET-B) found in smooth muscle cells of the vessel wall. The proliferative effect of ET-1to these cells has been detected in *in vitro* studies. However, because of the presence of ET-A and ET-B receptors in endothelial cell membranes and fibroblasts, the expression of leukocyte adhesion molecules is elevating as a result of stimulation of these cells by ET-1. The prevention of neointima formation was possible by antogonizing of ET receptors and systemic inhibition of ET-1^14^. Diltiazem can prevent endothelial hyperplasia, detected in our study, has not been previously observed in any study.

As in our study, previous studies have also revealed that diltiazem can prevent vascular wall thickening and lumen obstruction by preventing smooth muscle tissue damage of vessel wall. A study on monkeys has highlighted that diltiazem successfully eliminated vasospasm in brain vessels^15^.

The concluded findings have also been elaborated on in studies with different experimental models. The main achievement of our study was to transpire the correlation between diltiazem and endothelial hyperplasia. This fact with morphological parameters may be very valuable in future studies with diltiazem. It can be conducive to coming up with more significant and solid findings in this respective area of observation.

## Conclusion

According to final findings of the study on rabbits with a developed model of ischemia, with prescription of diltiazem, endothelial hyperplasia on microcirculatory system of skin, the thickening of arterial vessel walls and the thrombosis in vessel’s passage were rarely observed through statistical differences. Diltiazem eliminates the factors, causes vessel occlusion and hypoxia as its sequence. 

## Funding

Azerbaijan Medical University supported the study. The financial source of the study was Central Research Laboratory of Azerbaijan Medical University.

## Conflict of Interest

 The authors declare that there is no conflict of interests.
